# Atypical Ulcerated Cutaneous Presentation of Diffuse Large B-Cell Lymphoma: A Case Report

**DOI:** 10.7759/cureus.107974

**Published:** 2026-04-29

**Authors:** Paula Nogueira, Isabel V Rodrigues, Pedro Reboredo, Nataliya Pylyp, Mihail Mogildea

**Affiliations:** 1 Internal Medicine, Unidade Local de Saúde do Algarve - Hospital de Faro, Faro, PRT

**Keywords:** cutaneous lymphoma, diffuse large b-cell lymphoma, extranodal lymphoma, skin neoplasm, skin ulcer

## Abstract

Diffuse large B-cell lymphoma (DLBCL) is an aggressive malignancy that typically presents with nodal disease, while cutaneous involvement is uncommon and may lead to delayed diagnosis. We report the case of a 48-year-old man presenting with a rapidly enlarging ulcerated mass on the shoulder, complicated by hemorrhage and severe anemia. The lesion had progressed over six months and was associated with significant unintentional weight loss. Imaging revealed a necrotic soft tissue mass, and histopathological examination confirmed diffuse large B-cell lymphoma, centroblastic subtype, with a high proliferative index (Ki-67 70-80%). The patient’s clinical course was rapidly progressive, and he died before complete staging, including positron emission tomography (PET) imaging, could be performed. This case highlights the importance of early biopsy of atypical or rapidly progressive cutaneous lesions, as delayed recognition of extranodal presentations of aggressive lymphomas may adversely impact patient outcomes.

## Introduction

Diffuse large B-cell lymphoma (DLBCL) is the most common subtype of non-Hodgkin lymphoma, accounting for approximately 30-40% of cases worldwide, and is characterized by aggressive clinical behavior and heterogeneous presentation [1,2]. It predominantly affects older adults, with a slight male predominance, although it can occur across a wide age range.

Extranodal involvement occurs in up to 30-40% of patients with DLBCL and may involve virtually any organ, including the gastrointestinal tract, central nervous system, and skin [3]. Cutaneous manifestations are relatively uncommon and may be underrecognized, particularly when presenting as atypical or ulcerated lesions.

A key distinction exists between primary cutaneous DLBCL, which originates in the skin without evidence of systemic disease at diagnosis, and secondary cutaneous involvement, which reflects dissemination of systemic lymphoma and is generally associated with a more aggressive clinical course and poorer prognosis [4]. Differentiating between these entities is essential but may be challenging, especially in the absence of overt nodal disease at presentation.

Ulcerated and rapidly enlarging skin lesions can mimic infectious, inflammatory, or other neoplastic conditions, such as soft tissue sarcomas, leading to diagnostic delay [5]. This is particularly relevant in patients with risk factors for lymphoma, including immunosuppression, chronic inflammatory states, and certain viral infections such as Epstein-Barr virus (EBV) and human immunodeficiency virus (HIV) [6].

Early recognition of atypical extranodal presentations is critical given the aggressive nature of DLBCL, as delays in diagnosis may result in advanced-stage disease and adversely impact clinical outcomes.

## Case presentation

A 48-year-old man presented to the emergency department with severe asthenia and dyspnea on minimal exertion following an episode of profuse bleeding from a large ulcerated mass on the left shoulder. He reported a six-month history of rapid lesion growth, associated with significant unintentional weight loss (>10%), without fever or night sweats. Three months prior, computed tomography (CT) had identified a suspicious lesion in the left deltoid muscle. His past medical history included asthma. Social history was notable for heavy smoking (72 pack-years), chronic alcohol consumption (~210 g/day), and active heroin use. The patient lived alone, with limited social support.

**Figure 1:**
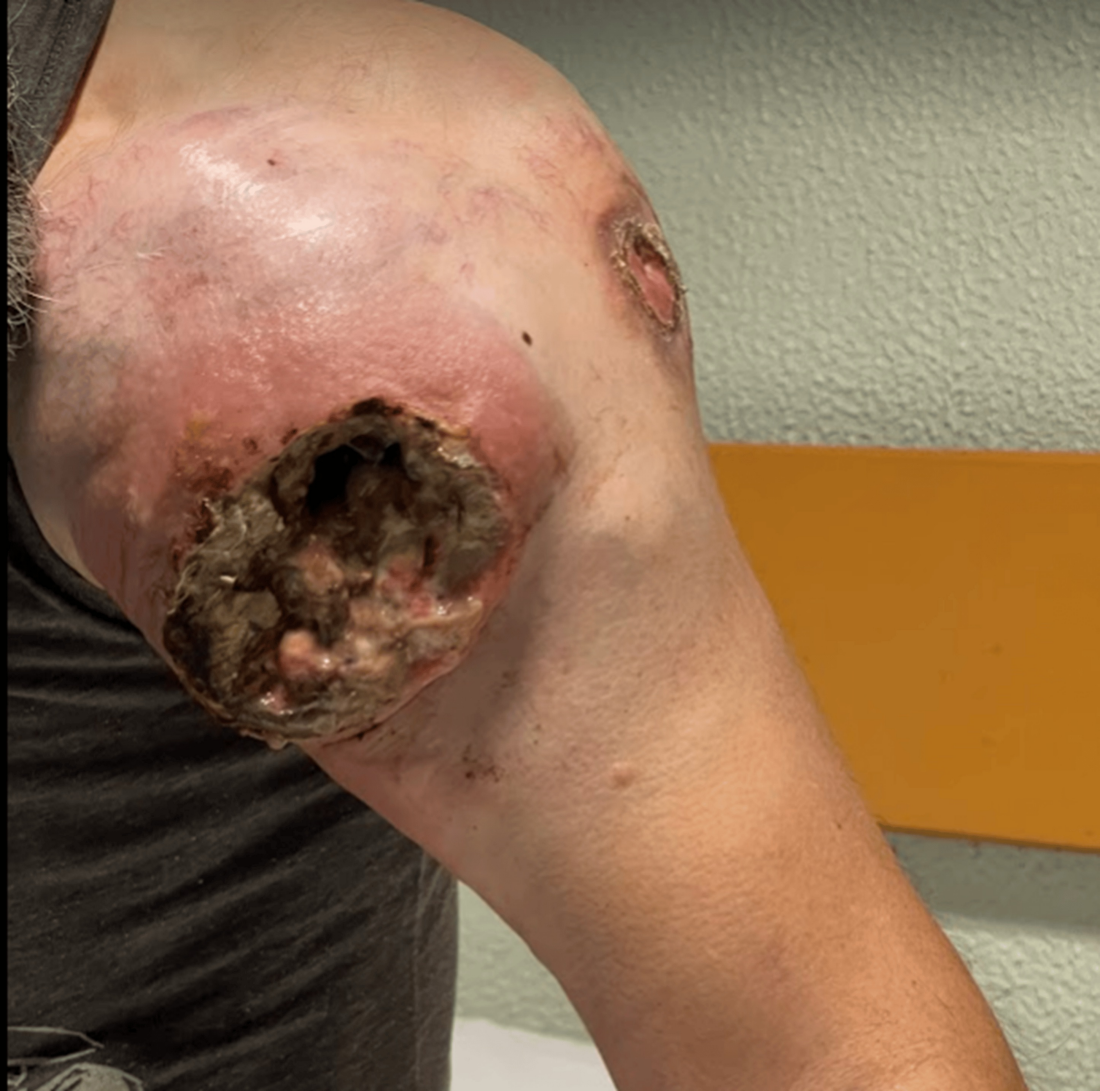
Ulcerated mass on the left shoulder demonstrating a necrotic base, exudate, and prominent neovascularization.

**Figure 2:**
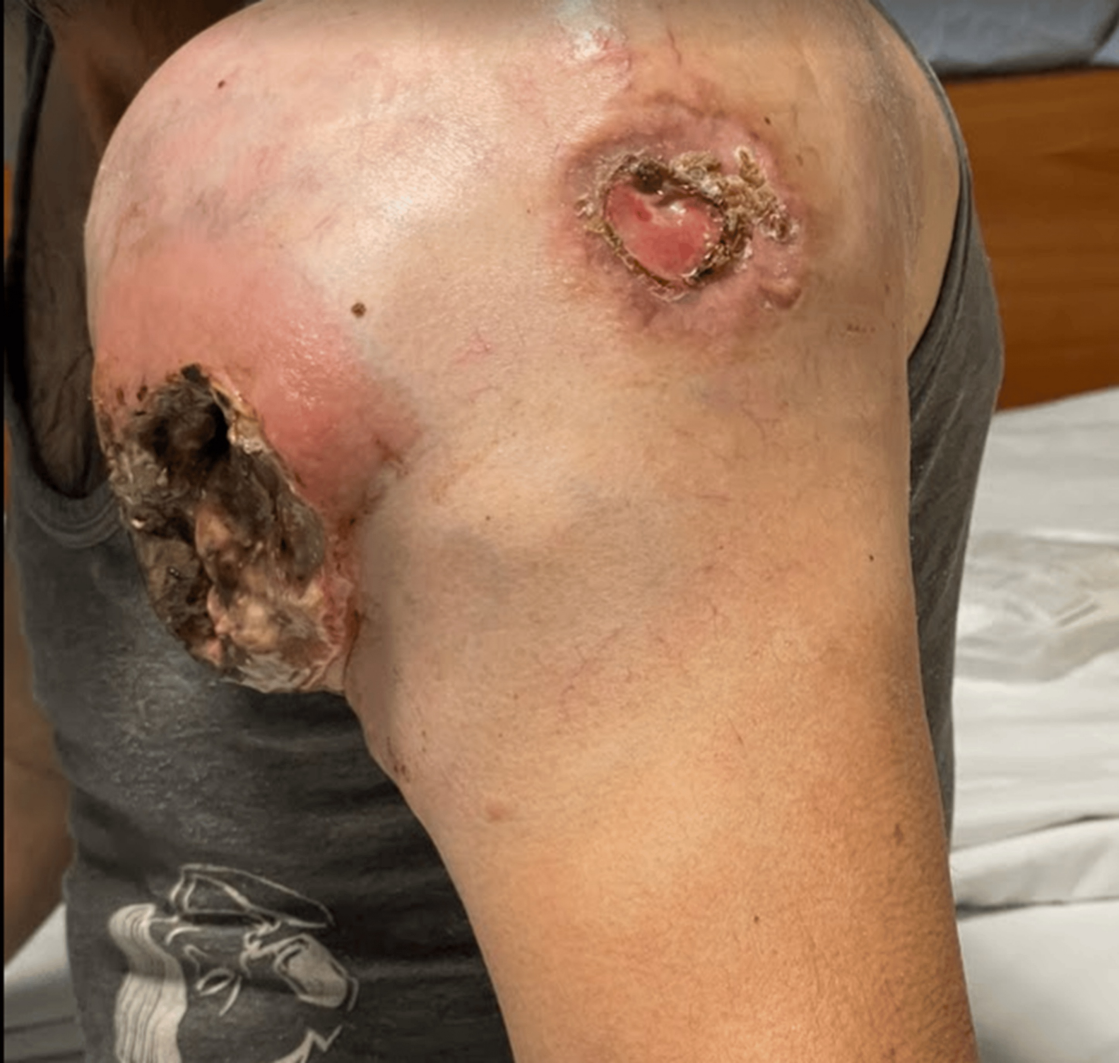
Ulcerated mass on the left shoulder (lateral view)

On physical examination, the patient appeared cachectic and in poor general condition. A large mass (~15 cm) was observed on the anterior left shoulder, with prominent neovascularization. An excavated ulcer with a necrotic and exudative base was present, with signs of recent bleeding and surrounding inflammation (Figures 1-2). There was marked edema of the entire left upper limb. No palpable lymphadenopathy was identified.

Laboratory investigations revealed severe anemia (hemoglobin 3.8 g/dL; reference range: 13-17 g/dL), leukocytosis (13 × 10⁹/L; reference range: 4-11 × 10⁹/L), thrombocytosis (545 × 10⁹/L; reference range: 150-400 × 10⁹/L), and elevated inflammatory markers (CRP 95 mg/L; reference range: <5 mg/L). Lactate dehydrogenase (LDH) levels were not available.

**Figure 3:**
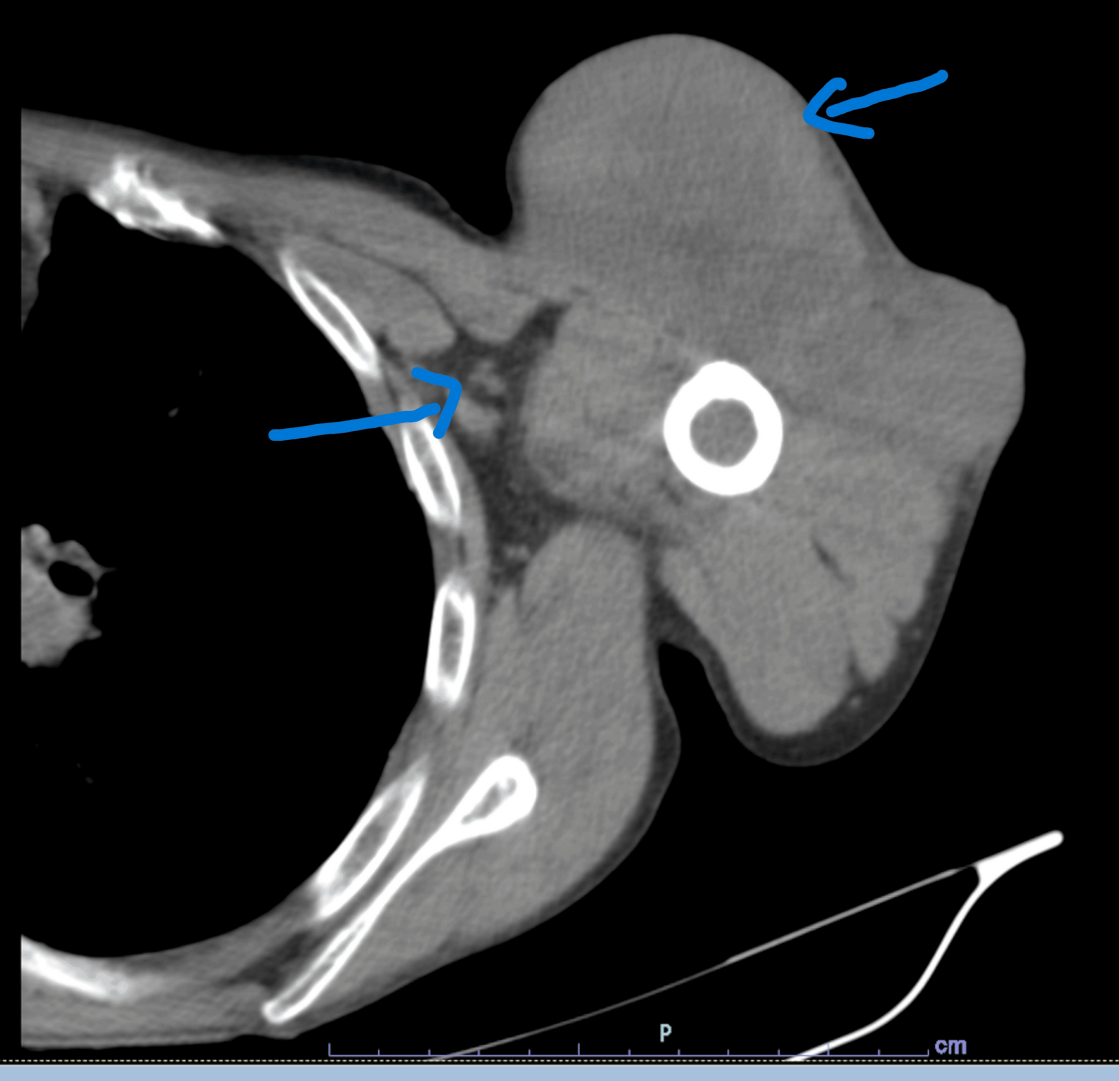
Axial CT image showing a necrotic lesion in the anterior deltoid muscle with peripheral enhancement.

**Figure 4:**
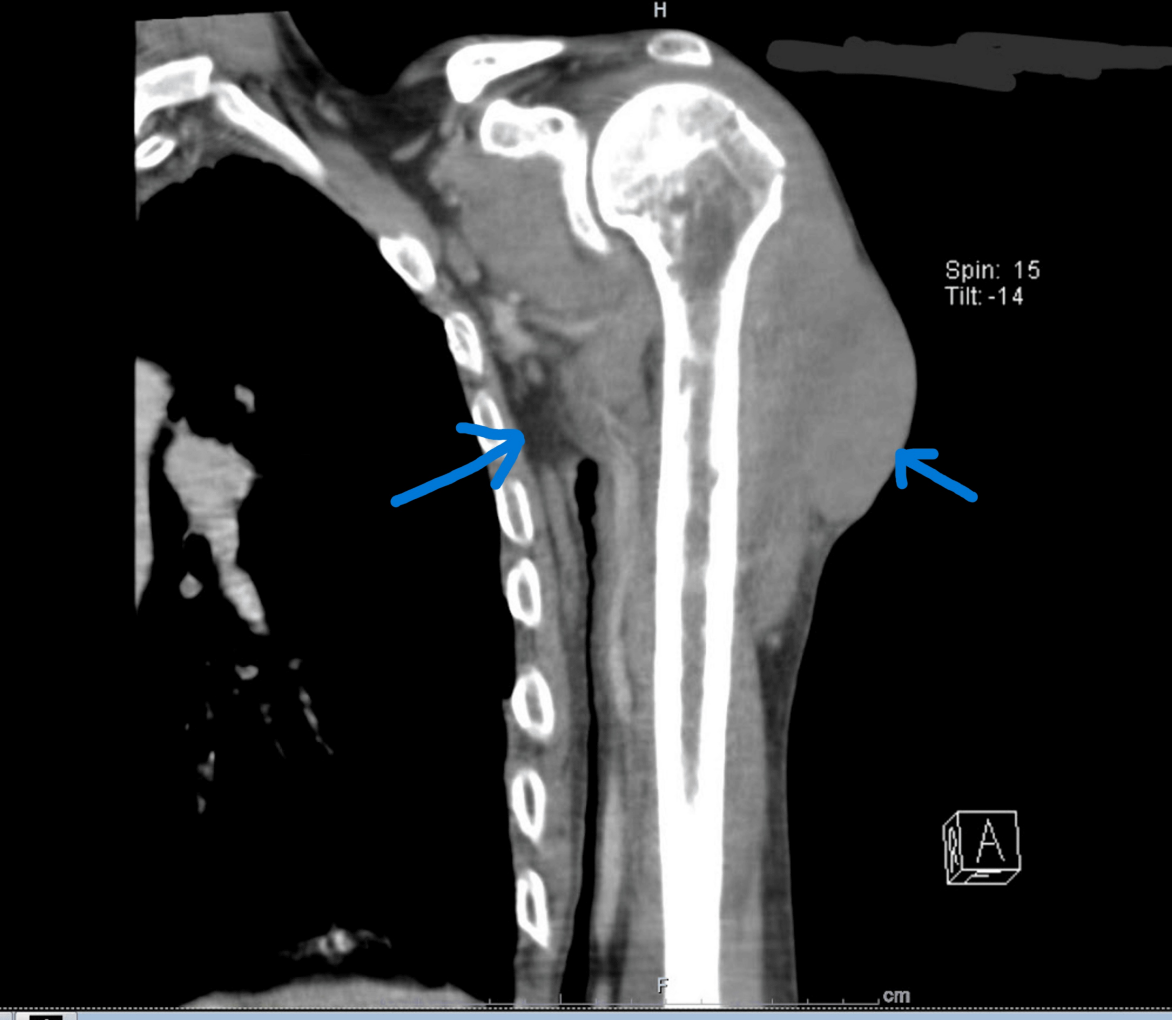
Coronal CT image demonstrating the extent of the lesion and associated regional involvement.

Initial CT imaging demonstrated a lesion in the anterior deltoid muscle measuring 7 × 5.8 cm, with peripheral enhancement and central necrosis (Figures 3-4). Staging CT revealed bilateral pulmonary ground-glass opacities suggestive of possible secondary involvement, mediastinal lymphadenopathy, and underlying emphysematous changes.

Given the rapid progression, ulceration, hemorrhagic complication, and unclear etiology of the lesion, a percutaneous biopsy was performed. The differential diagnosis at this stage included soft tissue sarcoma, chronic infectious process (including abscess), and metastatic malignancy. Histopathological examination revealed a poorly differentiated malignant neoplasm composed of large atypical lymphoid cells. Immunohistochemistry demonstrated positivity for CD45, CD20, BCL2, BCL6, CD10, and c-MYC, with a high Ki-67 proliferation index (70-80%), consistent with diffuse large B-cell lymphoma, centroblastic subtype [2,5].

The patient was referred to hematology for further management. A complete staging workup, including positron emission tomography (PET) imaging and bone marrow biopsy, was planned but could not be performed due to rapid clinical deterioration. Despite initial supportive management, the patient’s condition worsened significantly. Given the advanced disease and poor performance status at presentation, systemic therapy was not initiated. The patient died shortly after diagnosis.

## Discussion

Cutaneous involvement in diffuse large B-cell lymphoma (DLBCL) is uncommon and may represent either primary cutaneous lymphoma or secondary dissemination of systemic disease [3,6]. This distinction is clinically relevant, as primary cutaneous DLBCL generally follows a different biological behavior and prognosis compared to secondary cutaneous involvement, which is typically associated with more aggressive disease and systemic spread. However, differentiating between these entities can be challenging at presentation, particularly in the absence of significant lymphadenopathy or overt systemic findings [4].

Ulcerated cutaneous presentations are rare but have been associated with delayed diagnosis and more advanced disease at presentation [5]. These lesions are frequently misinterpreted as soft tissue sarcomas, chronic infections, or inflammatory processes due to their rapid growth, necrosis, and prominent inflammatory features [6]. Such diagnostic ambiguity may contribute to delays in tissue sampling and definitive diagnosis.

Cutaneous B-cell lymphomas typically present as nodules or plaques; however, ulceration has been linked to more aggressive clinical behavior and may reflect rapid tumor expansion and necrosis [5]. Secondary cutaneous involvement in systemic DLBCL more commonly presents with multifocal lesions, whereas solitary large ulcerated tumors, as observed in this case, are less frequently reported and may further complicate initial diagnostic assessment [6].

Common features reported across similar cases include rapid tumor growth, central necrosis, a high proliferative index (Ki-67 >70%), and delayed diagnosis due to atypical presentation [2]. A high Ki-67 index is a marker of increased cellular proliferation and has been associated with more aggressive disease and poorer clinical outcomes. In addition, co-expression of MYC and BCL2 proteins, referred to as “double expressor” lymphoma, is recognized as an adverse prognostic factor. This entity should be distinguished from “double hit” lymphoma, which is defined by chromosomal rearrangements involving MYC and BCL2 and/or BCL6 genes, typically identified by fluorescence in situ hybridization (FISH), and is associated with an even more aggressive clinical course and inferior survival outcomes [7].

Delayed diagnosis in aggressive lymphomas such as DLBCL is associated with advanced-stage disease at presentation, higher tumor burden, and worse prognosis. In the present case, the prolonged evolution of the lesion and its atypical ulcerated cutaneous presentation likely contributed to delayed recognition, ultimately limiting the ability to complete staging investigations, including positron emission tomography (PET) imaging and bone marrow biopsy, and to initiate timely treatment.

Extranodal lymphomas involving the skin and soft tissues pose significant diagnostic challenges, as imaging alone is insufficient to reliably distinguish lymphoma from other malignancies or infectious processes [6]. Therefore, early biopsy remains essential for accurate diagnosis and should be strongly considered in rapidly progressive or atypical cutaneous lesions, particularly when associated with necrosis, ulceration, or hemorrhagic complications.

Standard treatment for DLBCL typically consists of immunochemotherapy, most commonly rituximab combined with cyclophosphamide, doxorubicin, vincristine, and prednisone (R-CHOP), which has significantly improved survival outcomes in eligible patients [8]. Prognosis depends on several factors, including disease stage, performance status, and molecular features. However, in cases of advanced disease or poor clinical condition at presentation, as illustrated here, treatment may not be feasible.

The rapidly progressive course observed in this patient, culminating in early death prior to initiation of systemic therapy, underscores the aggressive nature of DLBCL with extranodal involvement and highlights the critical importance of early recognition. Atypical ulcerated cutaneous lesions, particularly when accompanied by hemorrhage and systemic symptoms, should prompt urgent diagnostic evaluation to avoid potentially fatal delays.

## Conclusions

Diffuse large B-cell lymphoma may rarely present as a large ulcerated cutaneous mass, mimicking infectious or soft tissue neoplastic processes and contributing to diagnostic delay. This case highlights the importance of maintaining a high index of suspicion for hematologic malignancies in atypical, rapidly evolving skin lesions. Ulceration and spontaneous hemorrhage should be recognized as important clinical red flags suggestive of aggressive underlying disease. Early biopsy and prompt histopathological evaluation are essential to establish the diagnosis and initiate appropriate management. Delayed recognition may result in advanced disease and poor clinical outcomes, as illustrated by the rapidly progressive course and fatal outcome in this case. Increased awareness of these uncommon presentations may help reduce diagnostic delays and improve patient outcomes.
